# Reduction of vascular reactivity in rat aortas following pilocarpine-induced status epilepticus

**DOI:** 10.1016/j.clinsp.2023.100195

**Published:** 2023-04-24

**Authors:** Karolini Zuqui Nunes, Fulvio Alexandre Scorza, Esper Abrão Cavalheiro, Dalton Valentim Vassallo

**Affiliations:** a Postgraduate Program in Nutrition and Health, Universidade Federal do Espírito Santo, Vitória, ES, Brazil; b Discipline of Neuroscience, Universidade Federal de São Paulo/Escola Paulista de Medicina, São PauloSP, Brazil; c Graduate Program in Physiological Sciences, Universidade Federal do Espírito Santo, Vitória, ES, Brazil

**Keywords:** Epilepsy, SUDEP, Aorta, Nitric Oxide, Hydrogen peroxide

## Abstract

**Objective:**

The authors investigated changes in vascular reactivity in rats following pilocarpine-induced status epilepticus.

**Method:**

Male Wistar rats weighing between 250g and 300g were used. Status epilepticus was induced using 385 mg/kg i.p. pilocarpine. After 40 days the thoracic aorta was dissected and divided into 4 mm rings and the vascular smooth muscle reactivity to phenylephrine was evaluated.

**Results:**

Epilepsy decreased the contractile responses of the aortic rings to phenylephrine (0.1 nM–300 mM). To investigate if this reduction was induced by increasing NO production with/or hydrogen peroxide L-NAME and Catalase were used. L-NAME (N-nitro-L arginine methyl ester) increased vascular reactivity but the contractile response to phenylephrine increased in the epileptic group. Catalase administration decreased the contractile responses only in the rings of rats with epilepsy.

**Conclusions:**

Our findings demonstrated for the first time that epilepsy is capable of causing a reduction of vascular reactivity in rat aortas. These results suggest that vascular reactivity reduction is associated with increased production of Nitric Oxide (NO) as an organic attempt to avoid hypertension produced by excessive sympathetic activation.

## Highlights

• Decreased phenylephrine-induced vascular reactivity.
• Increased production of NO.
• Increased in H2O2 production.

## Introduction

People with epilepsy may die suddenly and unexpectedly and without a clear underlying pathological etiology. This is defined as SUDEP (sudden unexpected death in epilepsy).[1] SUDEP is the main cause of lost years of life after a stroke.[1,2] Thus, a refined practice guideline has shown that SUDEP typically affects 1 in 4,500 children in 1 year and 1 in 1,000 adults with epilepsy per year.[3] Furthermore, several studies indicate that the major clinical risk factor for SUDEP is the presence or the number of Generalized Tonic-Clonic Seizures (GTCS).[3,4] The exact causes of SUDEP remain unknown.[2,5] However, experimental and clinical studies have strongly demonstrated that acute cardiovascular events during seizures (tachyarrhythmias, bradyarrhythmia's and ictal asystole), neurogenic pulmonary edema, respiratory disturbances with central and obstructive apnea, therefore caused by cardiorespiratory failure, have an important role for the occurrence of SUDEP.[6,7]

An additional hazardous fact is related to the status epilepticus which is the acute and intense activation of the sympathetic nervous system and the crosstalk between the Renin-Angiotensin System (RAS) and the sympathetic nervous system. Such occurrences might result in the development of hypertension, myocardial damage, and arrhythmogenic alterations that increase the sensibility for increment of ventricular arrhythmias, underlying mechanisms for SUDEP happening.[8,9] Cardiac alterations, hypertension and vascular dysfunction have been observed in epilepsy.[9,10] Vascular dysfunction is related to an increased risk of cardiovascular diseases and therefore, a vascular system might contribute to the emergence of such a situation.[11] Based on this, the aim of our study was to evaluate whether the pilocarpine-induced *status epilepticus* could affect the vascular reactivity of rats*.*

## Materials and methods

### Animals

All experiments were performed with male *Wistar* rats (250‒300g) and conducted in accordance with the guidelines for biomedical research as stated by the Brazilian Societies of Experimental Biology. The experimental protocol was approved by the Institutional Ethics Committee of the Federal University of Espírito Santo (CEUA 050/2015). This study followed the ARRIVE guidelines. All animals had free access to fed and water rat chow *ad libitum*. Rats were anesthetized with urethane 1.5 g/kg, i.p. and then sacrificed. The thoracic aortas were dissected, and the adipose tissue was removed. The aortas were divided into segments of 4 mm in length.

### Indunction of Status Epilepticus (SE)

To reduce the peripheral effects of pilocarpine, adult male Wistar rats (250‒300g) were given an i.p. injection of scopolamine methylnitrate (1 mg/kg; Sigma, St. Louis, MO) followed 30 min later by an i.p. injection of pilocarpine hydrochloride (385 mg/kg; Sigma) to induce SE. Seizure activity was monitored behaviorally and terminated with an i.p. injection of diazepam (10 mg/kg; Genéricos Hipolabor, Brasil) after 4h of convulsive SE. Only animals that displayed continuous, convulsive seizure activity after pilocarpine treatment were included in these studies. Control rats received the same injections of scopolamine methylnitrate and diazepam but received saline instead of pilocarpine. Vascular experimental protocols were performed 40 days after pilocarpine-induced SE.

### Vascular reactivity design

Aortic segments were mounted between two parallel wires in an organ bath containing Krebs-Henseleit solution (KHS, in mM: 124 NaCl, 4.6 KCl, 2.5 CaCl_2_, 1.2 MgSO_4_, 1.2 KH_2_PO_4_, glucose 11, 0.01 EDTA, and 23 NaHCO_3_, Ph 7.4) at 37°C under 95% O_2_-5% CO_2_. Aortic segments were put to an optimal resting tension of 1g, and the isometric tension was analyzed using a force transducer (TSD125C, CA, USA) attached to a data acquisition system (MP100A, BIOPAC System, Inc., Santa Barbara, USA) and connected to a computer.

After 45 min equilibration, aortic rings were initially exposed twice to 75 mM KCl. The first exposure assessed the functional integrity of the vessel, and the second exposure (30 min) assessed the maximal tension developed. This maneuver was used to define if there are no differences in contractions induced by KCl, which suggests that the voltage-operated channels in vascular smooth muscle were not affected, avoiding the effects of an additional variable. Afterward, endothelium viability was assessed with acetylcholine (10 µM) in segments that had been previously contracted with phenylephrine (1 µM). A relaxation equal to or greater than 90% was considered demonstrative of the integrity of the endothelium. Then, increasing concentrations of phenylephrine (10^−10^ to 3×10^−4^ M) were realized. A concentration–response curve for this contractile agonist was obtained, and the tension was measured when curve stabilization occurred on a plateau.

The function of endothelial-derived vasoactive factors on the phenylephrine-elicited contractile response was investigated. Aortic rings were incubated for 30 min with a nonspecific NOS inhibitor N_G_-nitro-L-arginine methyl ester (L-NAME, 100 μM) and catalase (1000 U/mL). Then, the phenylephrine concentration-response curves were obtained.

### Statistical analysis

The results are demonstrated as the mean ± standard error of the mean. Statistical analysis was performed using unpaired Student's *t*-test for the determination of maximum response values (Rmax); *p* < 0.05 values were defined as statistically significant. Differences in the Area Under the Concentration-response Curves (dAUC) for the control and experimental groups were used to compare the magnitude of effects and expressed as the percentage of the AUC of the corresponding control situation. Data analysis and figure generation were performed using the GraphPad Prism System (San Diego, CA, USA) and GB-STAT (Dynamic Microsystem Inc., Silver Spring, MD, USA). The values of “n” represent the number of animals used in each experimental protocol.

## Results

**Fig. 1:**
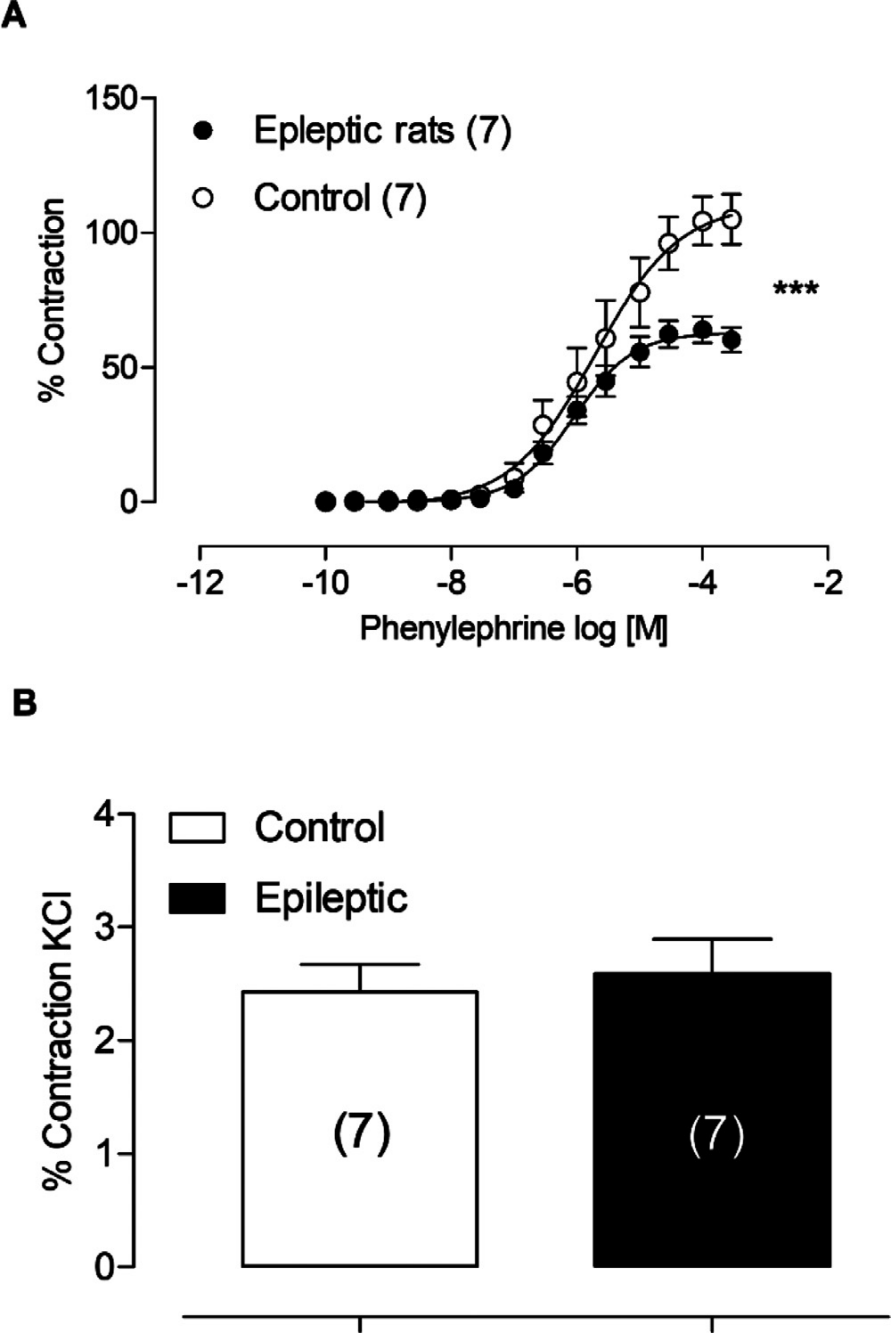
(A) Effects of curves to phenylephrine in aortic thoracic rings of epileptics and control rats. Each point represents the mean ± SEM. (B) Percentage of contraction to KCl in aortic thoracic rings of epileptic rats and controls. * *p* < 0.05 versus the corresponding control by Student's *t*-test. The amount of animals used is indicated in parentheses.

The results showed that the animals with epilepsy presented a reduction of the phenylephrine-induced contractions of rat aortas (Fig. 1A), however, no change was observed in relation to contraction with KCl, suggesting that in both groups the smooth muscle is intact (Fig. 1B).

**Fig. 2:**
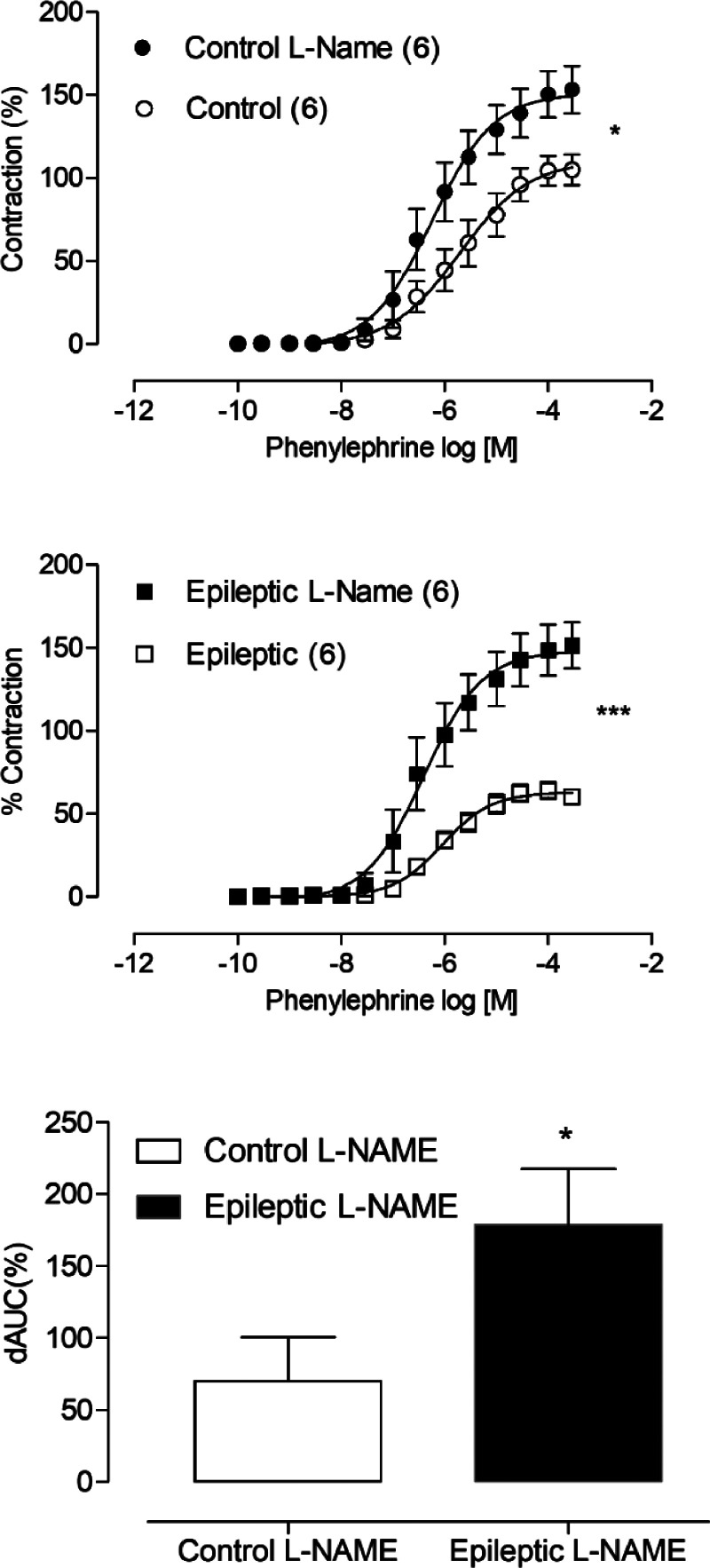
Effects of L-NAME (100 mM) in phenylephrine-induced vasoconstriction in aortic thoracic rings of epileptics and control rats. The graph show difference in the Area Under the Concentration-response curve (dAUC) in control and epileptic groups. * *p* < 0.05 versus the corresponding control by Student's *t*-test. The amount of animals used is indicated in parentheses.

With the aim of assessing whether epilepsy altered nitric oxide-induced modulation of phenylephrine-induced contractile responses, aortic thoracic rings were incubated with L-NAME (100 µM). The NOS inhibitor, L-NAME, increased the maximum response to phenylephrine in arteries from both groups (Fig. 2A). However, dAUC values demonstrated that the nitric oxide effect in the contractile response to phenylephrine was higher in the epileptic group (Fig. 2B).

**Fig. 3:**
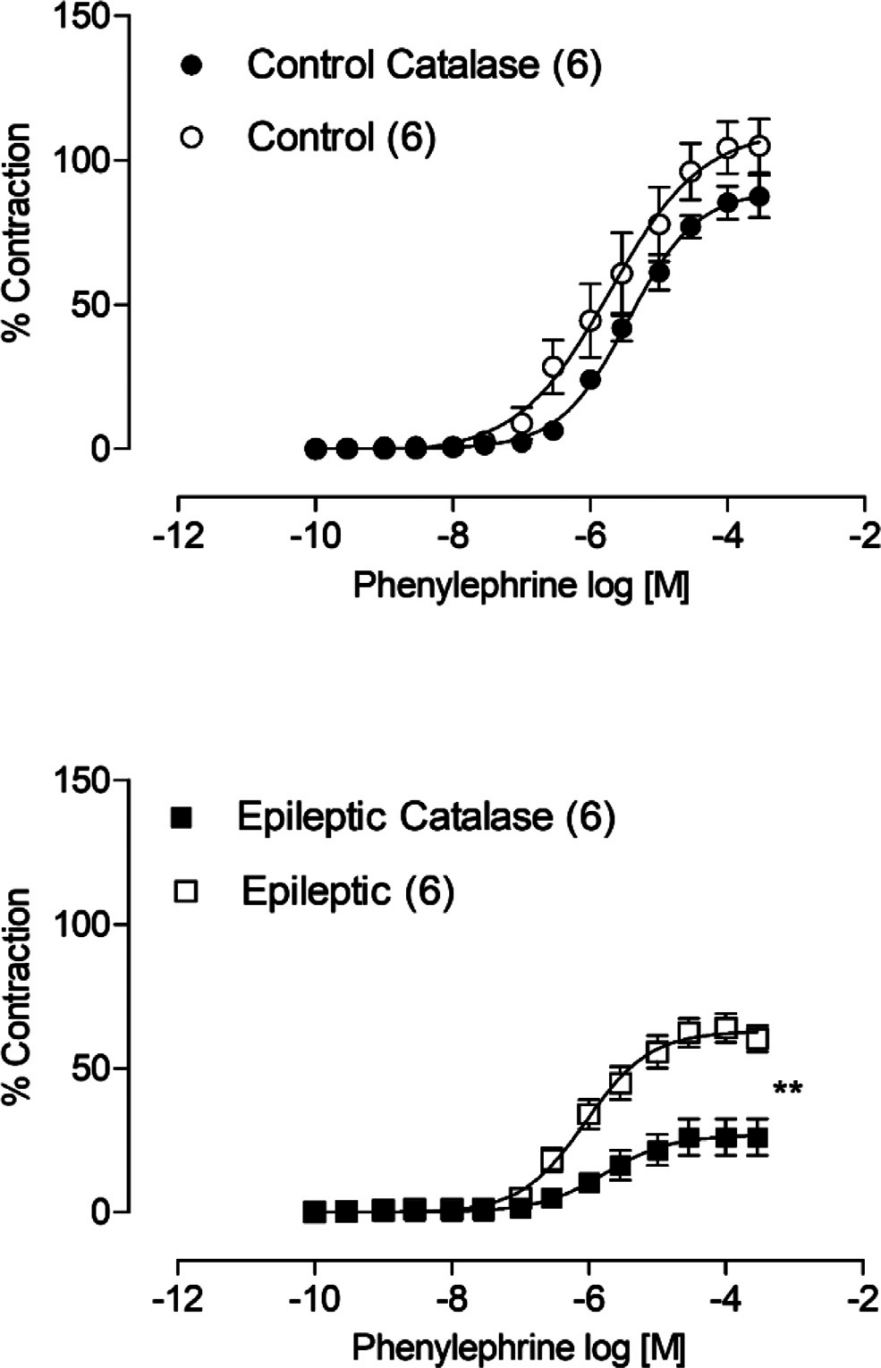
Effects of catalase (1000 U/mL) on the concentration-response curves to phenylephrine in aortic thoracic rings of epileptics and control rats. Each point represents the mean ± SEM.* *p* < 0.05 versus the corresponding control by Student's *t*-test. The amount of animals used is indicated in parentheses.

To investigate the participation of another vasodilator mechanism involved in the decrease of the contractile response to phenylephrine in the aortas thoracic rats with epilepsy, incubation with catalase, an enzyme capable of degrading H_2_O_2_, was performed. Catalase incubation decreased the vasoconstrictor response to phenylephrine only in rings from the epileptic group, suggesting an increase in the production of these free radicals, but producing a vasoconstrictor response in this group of animals (Fig. 3).

## Discussion

The authors demonstrate for the first time that epilepsy is capable of causing a reduction of vascular reactivity in rat aortas. Interestingly, this result is related to increased production of NO. On the other hand, the authors also observed an increase in H_2_O_2_ production acting as a vasoconstrictor. Therefore, the authors investigated the putative participation of the endothelium. In the vascular bed, an important mechanism that maintains blood vessels dilated is the endothelial NO Synthase (eNOS), which controls blood pressure and has numerous other vasoprotective and anti-atherosclerotic effects.[12] Our findings showed that this vasodilator mechanism involving NO plays an important role as suggested by the reduction of phenylephrine-induced contraction and the enhanced response to L-NAME in the epileptic group.

The change in vascular reactivity found in our study characterizes endothelial dysfunction in conductance vessels. Studies describe that endothelial dysfunction is also a feature present in cerebral vessels.[13] The neurovascular unit is unique to the brain and is a complex multicellular, functional anatomical structure that includes vascular cells and is related to the onset of seizures and epilepsy during aging. Authors suggest that treatments including anti-inflammatories and antioxidants aimed at restoring vascular functions may reduce or prevent seizures during aging.[14] Additionally, previous studies suggest an increased risk of vascular disorders in people with epilepsy, such as strokes, myocardial infarction, and peripheral vascular disease.[15] This suggests that previous vascular comorbidities might enhance vascular risks.

NO is involved in the signaling and regulation of virtually all critical cell functions, as well as a potent mediator of cell damage in a wide range of conditions. Recent evidence indicates that most of the cytotoxicity attributed to NO is due to peroxynitrite, produced from the diffusion-controlled reaction between NO and another free radical, the superoxide anion. These reactions trigger cellular responses ranging from subtle modulations of cell signaling to overwhelming oxidative damage, leading cells to necrosis or apoptosis.[16] In the central nervous system NO acts as a second messenger, neuromodulator, and neurotransmitter, which may suggest an essential role of this gaseous molecule in epilepsy and epileptogenesis.[17]

Epilepsy is the main cause of loss of years of life after stroke[1,2] because of SUDEP. This condition might result from the development of hypertension, myocardial damage, and arrhythmogenic alterations facts that are related to the status epilepticus linked to the activation of the sympathetic nervous system and the cross-talk between the Renin-Angiotensin System (RAS) and sympathetic nervous system.[8,9] Therefore, the participation of other components like the vascular system, should contribute to the development of SUDEP. Indeed, our results obtained from the evaluation of pilocarpine-induced *status epilepticus* in rats showed that the vascular reactivity was affected but instead of contributing to an increase in the hazardous occurrences, vascular reactivity reduced, suggesting an attempt to avoid those hazardous occurrences.

However, epilepsy is normally an episode that produces ischemia and in such conditions, Xanthine Oxidoreductase (XOR) might produce oxidative stress.[18] Under normal physiological conditions, H_2_O_2_ is the major O_2_-derived reactive product catalyzed by XOR reduction. However, in situations of ischemia H_2_O_2_ formation is also more favored.[19] Although the production of these radicals happens many times as a compensatory mechanism, the increase in their production leads to oxidative stress.[10] In addition, the authors must consider that H_2_O_2_ has a direct action on the vascular smooth muscle depending on its concentration. At low concentrations, H_2_O_2_ can act as a vasodilator and at higher concentrations as a vasoconstrictor.[20] Therefore, the authors investigated the putative participation of H_2_O_2_.

Both NO and H_2_O_2_ can act as vasodilator factors.[21,22] At first the authors believed that hydrogen peroxide could also be acting as a vasodilator, but our findings have shown the opposite results. Using catalase, an enzyme that degrades H_2_O_2_ into oxygen and water, the reactivity to phenylephrine was reduced in both groups, but more intensely in the epileptic group. These findings suggested that H_2_O_2_ was acting as a vasoconstrictor while NO was producing vasodilation. Since epilepsy produces hypoxic episodes[23,24] the authors believe that under hypoxia developed by epilepsy, an increased production H_2_O_2_ could occur. However, this mechanism was not enough to restrain the role played by NO in the contractile response to phenylephrine that reduced the vascular reactivity in the epileptic group mainly if previous conditions enhance hypertension development.

Our results demonstrate an increase in the bioavailability of NO and H_2_O_2_, which characterizes, in addition to endothelial dysfunction, an increase in oxidative stress. Oxidative stress and excessive reactive oxygen species production have been implicated in many neurological pathologies, including epilepsy. And they may be associated with DNA damage, lipid peroxidation, and protein oxidation, contributing to the emergence of comorbidities associated with epilepsy and the emergence of SUDEP.[25]

At the moment, it is well established that oxidative stress plays a key role in acute neurological injuries such as prolonged seizures.[26] The inhibition of NADPH oxidase, for example, in a model of Temporal Lobe Epilepsy induced by pilocarpine, demonstrated that the free radicals produced by NADPH oxidase are involved in the neurodegeneration induced by SE and suggested therapeutic interventions aimed at inhibiting this enzyme, may present results promising.[27]

Studies will be needed to understand the pathways related to decreased vascular reactivity, as well as to the increased bioavailability of hydrogen peroxide and nitric oxide. However, it is a fact that epilepsy causes a situation of oxidative stress and a breakdown of vascular homeostasis. Understanding and identifying the main mechanisms involved in vascular changes associated with epilepsy can provide prophylactic treatment to prevent the development of complications and SUDEP.

### Limitations

The main limitations of the study are related to the lack of investigation of other pathways involved in changing vascular reactivity.

### Future directions

Studies will be needed to describe other possible pathways involved in changing vascular reactivity. Investigating the formation of free radicals through the action of NADPH and mitochondria may be useful for understanding and detailing the possible pathways involved in changing vascular reactivity. Increased production of superoxide anions, for example, can result in interaction with nitric oxide, and these reactions trigger cellular responses that range from subtle modulations of cell signaling to overwhelming oxidative injury, leading cells to necrosis or apoptosis. Thus, new pharmacological and non-pharmacological strategies aimed at reducing oxidative stress may represent powerful therapeutic tools in the future.

## Conclusion

The authors demonstrated for the first time that epilepsy is capable of causing vascular tone reduction associated with increased production of nitric oxide and increased free radical production blunting cardiovascular homeostatic mechanisms. These results may be associated with vascular complications e associated with SUDEP, in addition to characterizing an increase in oxidative stress. Therefore, an analysis of endothelial function in patients at high risk of SUDEP may be beneficial.

## Authors' contributions

Karolini Zuqui Nunes: Study planning; development of experiments; data analysis; writing the manuscript; Original draft; Investigation; Formal analysis; Data curation

Fulvio Alexandre Scorza: Study planning; experimental model development; data analysis; writing the manuscript; Writing - review; Validation

Esper Abrão Cavalheiro: Study planning; experimental model development; data analysis; writing the manuscript; Conceptualization; Validation

Dalton Valentim Vassallo: Study planning; development of experiments; data analysis; writing the manuscript;Conceptualization; Funding acquisition; Formal analysis; Supervision

## Conflicts of interest

The authors declare no conflicts of interest.
